# Osteoporosis and Coronary Artery Disease Burden in Older Adults with Chronic Coronary Syndrome: A Two-Center Cross-Sectional Study in Vietnam

**DOI:** 10.3390/jcm15187009

**Published:** 2026-09-10

**Authors:** Tan Van Nguyen, Le Thi Ho, Linh Khanh Thi Vu, Huy Quang Nguyen

**Affiliations:** 1 Department of Geriatrics and Gerontology, University of Medicine and Pharmacy at Ho Chi Minh City, Ho Chi Minh City 700000, Vietnam; drhole87@gmail.com (L.T.H.);; 2 Comprehensive Geriatrics and Older Adults Leading Research Group (Code: 003.TCM2026), University of Medicine and Pharmacy at Ho Chi Minh City, Ho Chi Minh City 700000, Vietnam; 3 Department of Interventional Cardiology, Thong Nhat Hospital, Ho Chi Minh City 700000, Vietnam; 4 Department of Cardiology, Rheumatology, Endocrinology, Military Hospital 175, Ho Chi Minh City 700000, Vietnam; vuthikhanhlinh11547@gmail.com

**Keywords:** osteoporosis, coronary artery disease, chronic coronary syndrome, coronary angiography, Gensini score, older adults

## Abstract

**Background/Objectives:** Osteoporosis and coronary artery disease coronary artery disease (CAD) share common risk factors and biological pathways, yet their relationship in older adults with chronic coronary syndrome (CCS) remains incompletely understood. This study investigated the association between dual-energy X-ray absorptiometry (DXA)-defined osteoporosis and angiographic CAD burden in older adults with CCS. **Methods:** This two-center cross-sectional study included 451 consecutive adults aged ≥60 years with CCS who underwent DXA and invasive coronary angiography between July 2023 and June 2024. Osteoporosis was defined according to the World Health Organization criteria. CAD burden was assessed by disease extent (≥2-vessel or left main disease and ≥3-vessel or left main disease) and angiographic severity using the Gensini score. Multivariable logistic regression was performed after adjustment for age, sex, body mass index, hypertension, diabetes mellitus, chronic kidney disease, and smoking history. **Results:** Osteoporosis was identified in 151 participants (33.5%). Compared with participants without osteoporosis, those with osteoporosis had significantly higher prevalences of ≥2-vessel or left main disease (79.5% vs. 55.7%, *p* < 0.001), ≥3-vessel or left main disease (66.9% vs. 33.3%, *p* < 0.001), and severe CAD (Gensini score > 54; 29.0% vs. 15.0%, *p* = 0.022). After multivariable adjustment, osteoporosis remained independently associated with ≥2-vessel or left main disease (adjusted odds ratio [OR] 3.97, 95% confidence interval [CI] 2.31–6.81), ≥3-vessel or left main disease (adjusted OR 5.07, 95% CI 3.06–8.41), and severe CAD (adjusted OR 3.08, 95% CI 1.50–6.36; all *p* ≤ 0.002). **Conclusions:** DXA-defined osteoporosis was independently associated with a substantially greater angiographic burden of coronary artery disease in older adults with CCS. These findings suggest that osteoporosis may serve as a marker of more advanced coronary atherosclerosis and support closer cardiovascular risk assessment in this high-risk population.

## 1. Introduction

As populations continue to age, chronic coronary syndrome (CCS) and osteoporosis are increasingly encountered in the same patient. Coronary artery disease (CAD), the principal pathological substrate of CCS, remains a major cause of morbidity and mortality despite substantial advances in cardiovascular prevention, diagnosis, and treatment [1]. Osteoporosis is traditionally regarded as a skeletal disorder characterized by reduced bone mineral density (BMD), deterioration of bone strength, and an increased risk of fragility fractures. However, low BMD has also been associated with cardiovascular events and cardiovascular mortality [2]. A systematic review and meta-analysis further suggested that adults with low BMD had a higher prevalence of CAD. However, the strength of this association was attenuated in analyses that adjusted for shared cardiovascular risk factors [3].

The coexistence of osteoporosis and atherosclerotic cardiovascular disease may not be explained by aging alone. Bone loss and vascular disease share several biological processes, including chronic inflammation, oxidative stress, disturbances in calcium–phosphate metabolism, and age-related changes in cellular mineralization. Regulatory pathways involved in bone remodeling may also contribute to the osteogenic transformation of vascular smooth muscle cells and the development of vascular calcification. This interaction, commonly described as the bone–vascular axis, suggests that osteoporosis may reflect broader disturbances in biological aging and vascular health rather than an isolated disorder of the skeleton [4].

Clinical findings regarding the relationship between BMD and coronary atherosclerosis nevertheless remain inconsistent. The pooled association between low BMD and CAD reported in a previous meta-analysis was significant in the overall analysis, but was no longer conclusive when only multivariable-adjusted estimates were considered [3]. Similar uncertainty has been observed in studies of coronary artery calcification. A systematic review and meta-analysis found that patients with low BMD had a higher prevalence and greater extent of coronary artery calcification in unadjusted analyses. However, the association was substantially attenuated after adjustment for age and other potential confounders [5]. These results indicate that the observed relationship may depend on the skeletal site assessed, the definition of low BMD, the coronary imaging method, and the characteristics of the study population.

Evidence based on invasive coronary angiography is particularly limited. In patients with stable angina, lower BMD at the femoral neck and total hip was associated with multivessel CAD and a greater angiographic plaque burden assessed using a modified Gensini score [6]. A study of postmenopausal women similarly found that low femoral-neck BMD was independently associated with more severe coronary lesions [7]. In contrast, a larger study of men undergoing coronary angiography found no independent association between osteopenia or osteoporosis and either the presence of CAD or significant coronary stenosis [8]. Differences in sex, age distribution, sample size, clinical presentation, BMD measurement site, and angiographic definitions may partly explain these conflicting findings.

Relatively few studies have specifically examined older adults with established CCS using both dual-energy X-ray absorptiometry and invasive coronary angiography. Importantly, previous studies have largely focused on the presence of CAD or selected measures of angiographic severity, whereas evidence simultaneously characterizing both the anatomical extent and overall angiographic severity of CAD in older adults with established CCS remains limited. Therefore, the present study investigated the association between DXA-defined osteoporosis and the angiographic burden of CAD in older adults with CCS, using complementary measures of vessel involvement and Gensini score.

## 2. Materials and Methods

### 2.1. Study Design and Participants

This two-center cross-sectional study investigated the association between osteoporosis and the burden of coronary artery disease in older adults with chronic coronary syndrome. The study was conducted at the outpatient cardiology clinics of Military Hospital 175 and Thong Nhat Hospital, Ho Chi Minh City, Vietnam, between July 2023 and June 2024. Consecutive eligible patients were prospectively enrolled during the study period to minimize selection bias. DXA assessment was prospectively performed as part of the study protocol in all enrolled participants and was not based on a pre-existing clinical indication for DXA.

Patients were eligible if they were aged ≥60 years and had chronic coronary syndrome (CCS) with clinically stable symptoms, consistent with the framework for stable chest pain and known coronary artery disease described in the 2021 AHA/ACC/ASE/CHEST/SAEM/SCCT/SCMR Guideline for the Evaluation and Diagnosis of Chest Pain [9]. For the present study, eligibility additionally required angiographically documented coronary artery disease, defined as ≥50% luminal diameter stenosis in at least one major epicardial coronary artery on invasive coronary angiography (ICA). All participants were required to have undergone ICA within 12 months before study enrollment. In addition, only patients who had not previously received anti-osteoporotic treatment were included.

A total of 451 participants fulfilled all eligibility criteria and were included in the final analysis. The required sample size was estimated using the formula for estimating a single population proportion. Based on a previously reported osteoporosis prevalence of 36% among older adults with coronary artery disease, a two-sided confidence level of 95% (Z = 1.96), and a margin of error of 5%, the minimum required sample size was 354 participants [10]. Therefore, the final sample exceeded the calculated minimum sample size.

### 2.2. Clinical Assessment

Baseline demographic characteristics, cardiovascular risk factors, anthropometric measurements, medical history, medication use, and comorbidities were collected using standardized interviews, physical examination, and review of electronic medical records. Body mass index (BMI) was calculated as weight divided by height squared (kg/m^2^).

Hypertension, diabetes mellitus, dyslipidemia, chronic kidney disease, heart failure, and chronic obstructive pulmonary disease were diagnosed according to contemporary international guideline recommendations or documented physician diagnoses in the medical records [11,12,13,14,15].

### 2.3. Bone Mineral Density Assessment

Bone mineral density was measured at the lumbar spine (L1–L4) and femoral neck using dual-energy X-ray absorptiometry (Discovery™, Hologic Inc., Marlborough, MA, USA; Model X762C) at both participating centers. BMD values were expressed in grams per square centimeter (g/cm^2^). Osteoporosis was defined as a T-score ≤ −2.5 at either the lumbar spine or femoral neck, consistent with established densitometric diagnostic criteria for postmenopausal women and men aged ≥50 years [16,17].

### 2.4. Coronary Artery Disease Assessment

Coronary anatomy was evaluated using invasive coronary angiography performed within 12 months before study enrollment. Coronary imaging was interpreted by experienced cardiologists at the participating institutions according to contemporary clinical practice. The extent of established coronary artery disease was assessed according to documented involvement of the major epicardial coronary vessels and categorized as one-vessel, two-vessel, three-vessel, or left main coronary artery disease. Significant CAD was defined as ≥50% luminal diameter stenosis. In participants with previous coronary revascularization, available angiographic and revascularization records were additionally reviewed when determining the documented extent of coronary artery involvement.

The overall burden of coronary atherosclerosis was quantified using the Gensini scoring system, a validated angiographic scoring method that incorporates both the severity of luminal narrowing and the anatomical importance of each coronary segment. Briefly, stenosis severity was assigned weighted scores according to the percentage of luminal narrowing (1 for 1–25%, 2 for 26–50%, 4 for 51–75%, 8 for 76–90%, 16 for 91–99%, and 32 for total occlusion), which were subsequently multiplied by location-specific weighting factors reflecting the functional significance of each coronary segment. The final Gensini score was calculated as the sum of all weighted lesion scores, with higher scores indicating a greater coronary atherosclerotic burden [18,19]. For descriptive analyses, CAD burden was categorized as mild (Gensini score 0–23), moderate (24–54), or severe (>54) according to previously published studies [19].

### 2.5. Statistical Analysis

Statistical analyses were performed using IBM SPSS Statistics for Windows, version 27.0 (IBM Corp., Armonk, NY, USA). Continuous variables were assessed for normality using the Shapiro–Wilk test and are presented as mean ± standard deviation (SD) or median with interquartile range (IQR), as appropriate. Categorical variables are presented as frequencies and percentages.

Baseline demographic characteristics, clinical variables, and coronary artery disease characteristics were compared between participants with and without osteoporosis. Normally distributed continuous variables were compared using Welch’s independent-samples *t*-test, whereas non-normally distributed continuous variables were compared using the Mann–Whitney U test. Categorical variables were compared using Pearson’s chi-square test or Fisher’s exact test, as appropriate.

Univariable and multivariable binary logistic regression analyses were performed to evaluate the association between osteoporosis and coronary artery disease burden. Osteoporosis status was the primary exposure, with participants without osteoporosis serving as the reference group. Three prespecified binary outcomes were examined: (1) ≥2-vessel or left main coronary artery disease, (2) ≥3-vessel or left main coronary artery disease, and (3) severe coronary artery disease, defined as a Gensini score > 54. Multivariable models were adjusted for age, sex, body mass index, hypertension, diabetes mellitus, chronic kidney disease, and smoking history. These covariates were selected a priori based on their clinical relevance and potential roles as confounders. Analyses were performed using complete-case data for each statistical model. Because Gensini scores were available for only 266 participants, analyses of severe coronary artery disease (Gensini score > 54) were restricted to this subgroup. In an exploratory analysis, angiographic CAD burden was additionally compared across the three WHO BMD categories (normal BMD, osteopenia, and osteoporosis). Results are presented as crude and adjusted odds ratios (ORs) with corresponding 95% confidence intervals (CIs). Statistical significance was defined as a two-sided *p* value < 0.05.

### 2.6. Use of Generative Artificial Intelligence

During the revision of this manuscript, the authors used ChatGPT (OpenAI, GPT-5.6 Sol) to assist with language refinement, manuscript organization, and consistency checking of the text, tables, and figures. All AI-assisted outputs were critically reviewed, verified, and edited by the authors. ChatGPT was not used to generate or modify the original study data.

### 2.7. Ethics

The study was conducted in accordance with the ethical principles of the Declaration of Helsinki. The study protocol was approved by the Ethics Committee of the University of Medicine and Pharmacy at Ho Chi Minh City (Approval No. 678/HĐĐĐ-ĐHYD, dated 20 July 2023) and the Ethics Committee of Thong Nhat Hospital (Approval No. 71/2023/BVTN-HĐYĐ, dated 24 July 2023). Written informed consent was obtained from all participants prior to enrollment. Participant confidentiality was strictly maintained by anonymizing all data before analysis, and the collected information was used exclusively for research purposes.

## 3. Results

### 3.1. Study Population

**Figure 1:**
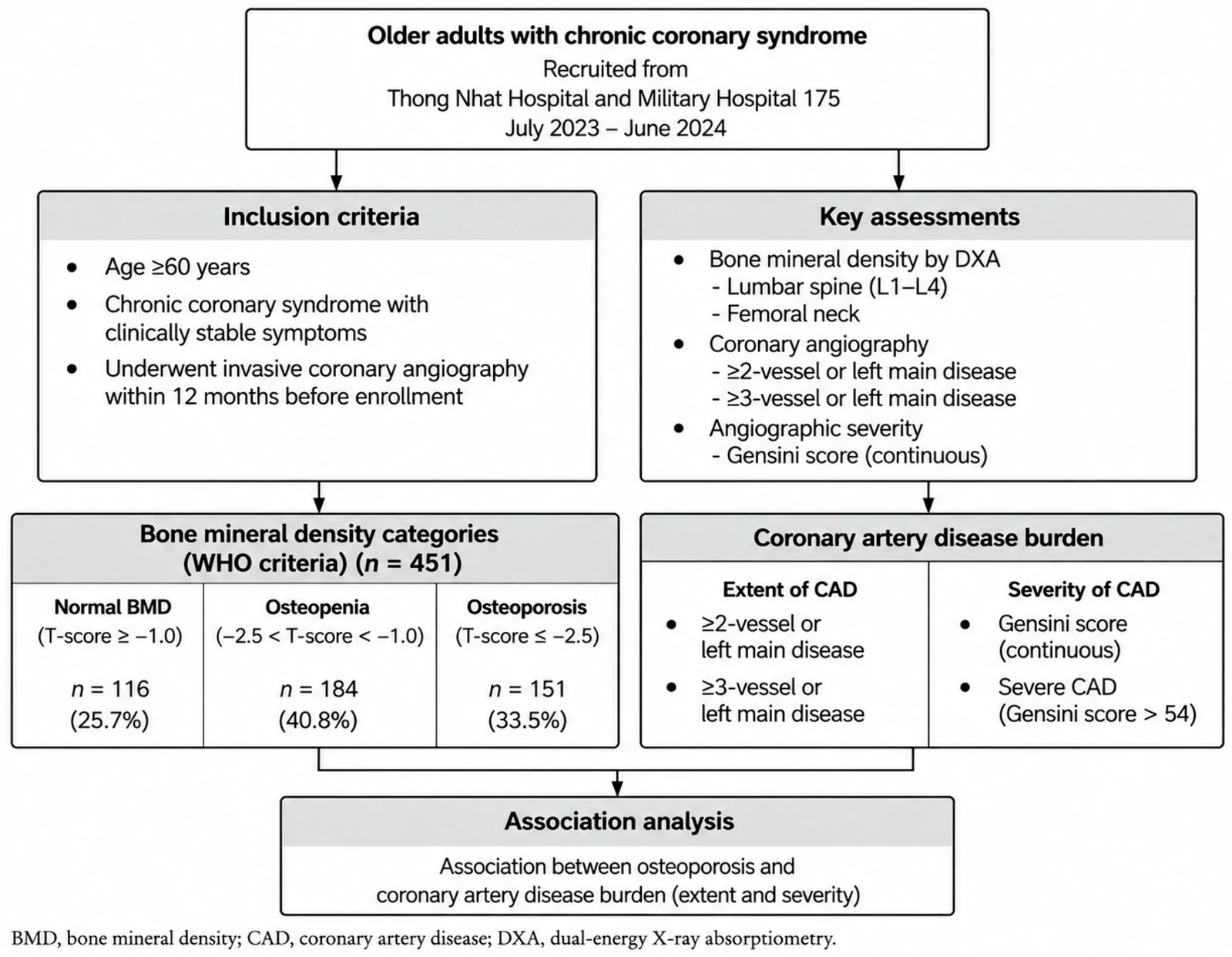
Study design and participant flow. Older adults with chronic coronary syndrome recruited from Thong Nhat Hospital and Military Hospital 175 between July 2023 and June 2024 underwent bone mineral density assessment by dual-energy X-ray absorptiometry (DXA) and invasive coronary angiography. Bone mineral density was categorized according to the World Health Organization criteria. Coronary artery disease burden was assessed using prespecified measures of disease extent (≥2-vessel or left main disease and ≥3-vessel or left main disease) and angiographic severity based on the Gensini score, which was available for 266 participants.

During the study period, 451 older adults with chronic coronary syndrome met the eligibility criteria and were included in the final analysis. The mean age was 70.4 ± 7.1 years, 297 participants (65.9%) were men, and 151 (33.5%) had osteoporosis. Among the remaining participants, 184 (40.8%) had osteopenia and 116 (25.7%) had normal bone mineral density. The study design and participant flow are shown in Figure 1.

### 3.2. Baseline Characteristics According to Osteoporosis Status

Baseline characteristics of the study population according to osteoporosis status are presented in Table 1. Compared with participants without osteoporosis, those with osteoporosis were significantly older (72.0 ± 7.4 vs. 69.6 ± 6.8 years, *p* < 0.001), were less likely to be male (37.7% vs. 80.0%, *p* < 0.001), and had a lower body mass index (22.6 ± 2.9 vs. 24.0 ± 2.8 kg/m^2^, *p* < 0.001). They were also less likely to have a smoking history (8.6% vs. 20.7%, *p* = 0.001) and to engage in regular physical activity (64.2% vs. 78.7%, *p* = 0.001).

With respect to clinical characteristics, the prevalence of hypertension, diabetes mellitus, dyslipidemia, chronic kidney disease, heart failure, chronic obstructive pulmonary disease, and alcohol consumption did not differ significantly between the two groups. In contrast, previous fracture was significantly more common among participants with osteoporosis than among those without osteoporosis (15.9% vs. 8.7%, *p* = 0.021).

Regarding the comprehensive geriatric assessment, participants with osteoporosis had significantly higher prevalences of frailty (53.0% vs. 25.7%, *p* < 0.001), malnutrition or nutritional risk (15.9% vs. 7.7%, *p* = 0.007), impairment in activities of daily living (19.9% vs. 7.3%, *p* < 0.001), impairment in instrumental activities of daily living (51.0% vs. 25.0%, *p* < 0.001), and cognitive impairment (19.2% vs. 11.7%, *p* = 0.030).

### 3.3. Coronary Angiographic Characteristics 

Coronary angiographic characteristics are summarized in Table 2. Participants with osteoporosis exhibited a significantly greater burden of coronary artery disease than those without osteoporosis. The overall distribution of coronary artery disease extent differed significantly between the two groups (*p* < 0.001). Participants with osteoporosis had a markedly lower prevalence of one-vessel disease (21.2% vs. 46.3%) but substantially higher prevalences of three-vessel disease (46.4% vs. 23.3%) and left main disease (32.5% vs. 18.3%, *p* = 0.001). Consequently, both ≥2-vessel or left main disease (79.5% vs. 55.7%, *p* < 0.001) and ≥3-vessel or left main disease (66.9% vs. 33.3%, *p* < 0.001) were significantly more frequent among participants with osteoporosis.

Among the 266 participants with available Gensini scores, the median Gensini score was numerically higher in the osteoporosis group than in the non-osteoporosis group [28 (IQR 9–59) vs. 24 (IQR 10–44)], although the difference did not reach statistical significance (*p* = 0.130). However, the overall distribution of Gensini severity categories differed significantly between the two groups (*p* = 0.022), with severe coronary artery disease (Gensini score > 54) being nearly twice as common in participants with osteoporosis (29.0% vs. 15.0%).

In an exploratory analysis across the three WHO BMD categories, the prevalence of ≥2-vessel or left main disease was 59.5% in participants with normal BMD, 53.3% in those with osteopenia, and 79.5% in those with osteoporosis (overall *p* < 0.001). The corresponding prevalences of ≥3-vessel or left main disease were 37.1%, 31.0%, and 66.9%, respectively (overall *p* < 0.001). Among participants with available Gensini scores, severe CAD (Gensini score > 54) was present in 8.8%, 18.1%, and 29.0% of the normal-BMD, osteopenia, and osteoporosis groups, respectively (overall *p* = 0.009). Osteopenia did not differ significantly from normal BMD for ≥2-vessel or left main disease (*p* = 0.291), ≥3-vessel or left main disease (*p* = 0.276), or severe CAD (*p* = 0.106).

### 3.4. Association Between Osteoporosis and Coronary Artery Disease Burden

The associations between osteoporosis and coronary artery disease burden were evaluated using crude and multivariable logistic regression analyses, and the results are presented in Table 3. In the crude analyses, osteoporosis was significantly associated with higher odds of ≥2-vessel or left main disease, ≥3-vessel or left main disease, and severe coronary artery disease, defined as a Gensini score > 54.

After adjustment for age, sex, body mass index, hypertension, diabetes mellitus, chronic kidney disease, and smoking history, osteoporosis remained independently associated with ≥2-vessel or left main disease (adjusted OR 3.97, 95% CI 2.31–6.81; *p* < 0.001) and ≥3-vessel or left main disease (adjusted OR 5.07, 95% CI 3.06–8.41; *p* < 0.001). Among the 266 participants with available Gensini scores, osteoporosis was also independently associated with severe coronary artery disease (adjusted OR 3.08, 95% CI 1.50–6.36; *p* = 0.002).

**Figure 2:**
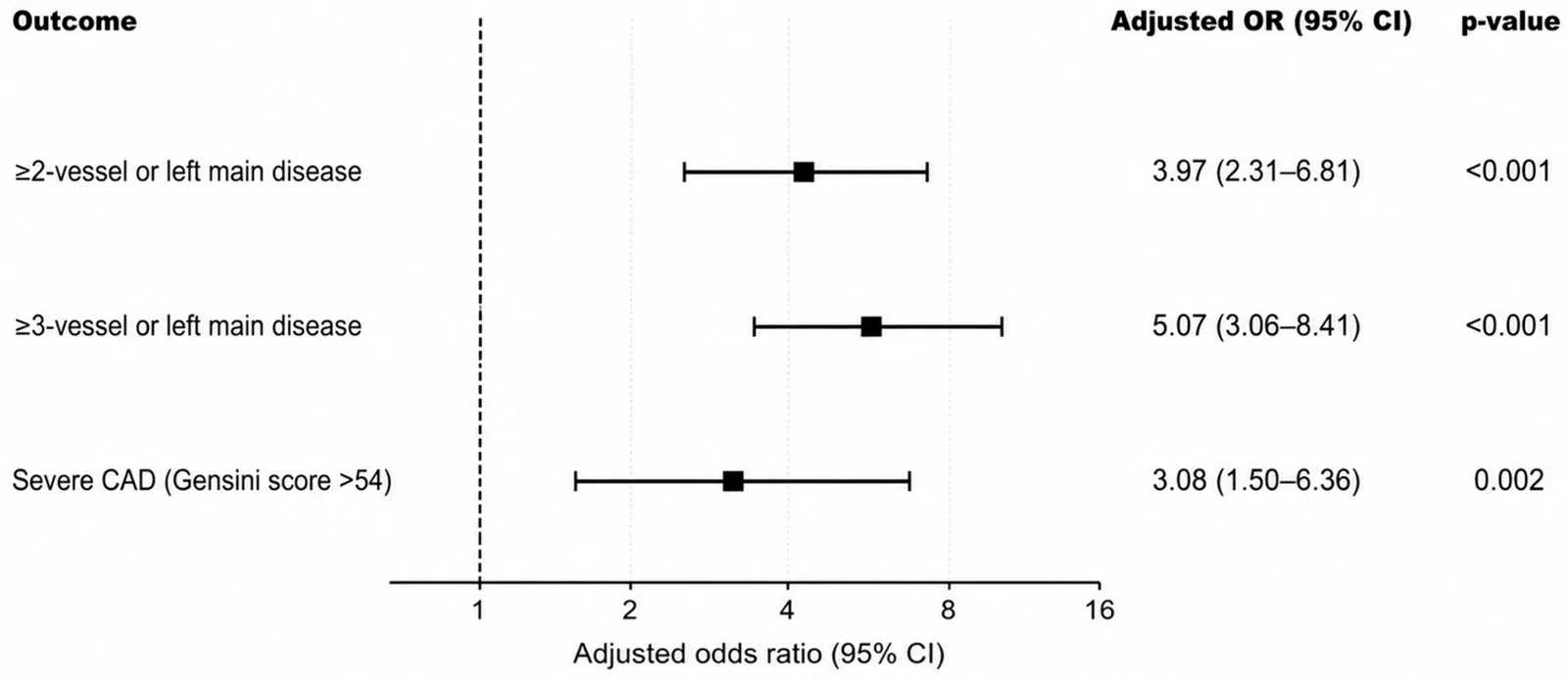
Association between osteoporosis and coronary artery disease burden. Forest plot showing adjusted odds ratios and 95% confidence intervals for the associations of osteoporosis with ≥2-vessel or left main disease, ≥3-vessel or left main disease, and severe coronary artery disease defined as a Gensini score > 54. Multivariable logistic regression models were adjusted for age, sex, body mass index, hypertension, diabetes mellitus, chronic kidney disease, and smoking history. The reference group was participants without osteoporosis. OR, odds ratio; CI, confidence interval.

Figure 2 illustrates the adjusted odds ratios and corresponding 95% confidence intervals, demonstrating the consistent independent association between osteoporosis and increasing coronary artery disease burden across all study outcomes.

## 4. Discussion

In this two-center study of older adults with chronic coronary syndrome, we found that DXA-defined osteoporosis was independently associated with a greater angiographic burden of coronary artery disease. Compared with participants without osteoporosis, those with osteoporosis were more likely to have extensive coronary involvement and severe coronary atherosclerosis, independent of conventional cardiovascular risk factors.

Our findings are consistent with, but quantitatively stronger than, much of the previous evidence linking low bone mineral density to coronary atherosclerosis. In the present study, osteoporosis was independently associated with a greater coronary artery disease burden. This association was observed across complementary measures of coronary artery disease burden, including both anatomical extent and angiographic severity, after adjustment for conventional cardiovascular risk factors.

These results extend the findings of the systematic review and meta-analysis by Khandkar et al., which included 10 observational studies and found that low bone mineral density was associated with coronary artery disease in the pooled analysis (OR 1.65, 95% CI 1.37–2.39) [3]. However, when the analysis was restricted to the four studies reporting multivariable-adjusted estimates, the association was attenuated and did not reach statistical significance (pooled adjusted OR 3.01, 95% CI 0.91–9.99; *p* = 0.07), indicating substantial uncertainty regarding the contribution of age and shared cardiovascular risk factors [3]. In contrast, the associations in our study remained significant after adjustment for age, sex, body mass index, hypertension, diabetes mellitus, chronic kidney disease, and smoking history.

The study by Guan et al. provides the most directly comparable angiographic evidence [6]. Among 459 patients with stable chest pain undergoing DXA and coronary angiography, osteoporosis at the femoral neck was independently associated with multivessel coronary artery disease (adjusted OR 4.34, 95% CI 2.05–9.20), while osteoporosis at the total hip was associated with an adjusted OR of 2.91 (95% CI 1.29–6.60). Lower femoral-neck and total-hip bone mineral density were also associated with greater angiographic disease severity, whereas no corresponding association was observed for lumbar-spine bone mineral density [6]. Overall, our findings are consistent with those of Guan et al., while extending the evidence by evaluating both anatomical extent and angiographic severity in an older population with chronic coronary syndrome.

Xu et al. reported a similar association in 122 postmenopausal women with angiographically confirmed coronary artery disease [7]. In that study, 85 participants (69.7%) had severe coronary lesions, defined as a Gensini score ≥ 25. The femoral-neck T-score was lower among women with severe coronary lesions than those with mild ones (−1.42 ± 1.39 vs. −0.84 ± 1.01), and femoral-neck osteopenia or osteoporosis was independently associated with severe disease (OR 2.73, 95% CI 1.06–6.13). The femoral-neck T-score was also independently and inversely related to the Gensini score (β = −0.407; *p* = 0.007) [7]. Although their Gensini threshold was lower than ours and their study included only postmenopausal women, the magnitude of association was broadly comparable with the adjusted OR of 3.08 for severe coronary artery disease in our cohort. In contrast, Beer et al. found no independent association between low bone mineral density and angiographically determined coronary atherosclerosis in 623 men undergoing coronary angiography [8]. Neither osteopenia nor osteoporosis was independently associated with the presence of coronary artery disease or significant coronary stenosis after multivariable adjustment [8]. This discrepancy may partly reflect differences in study populations and outcome definitions. Whereas Beer et al. evaluated the presence or absence of coronary artery disease, our study and that of Guan et al. assessed the anatomical extent and overall angiographic burden of disease, which may better discriminate differences in coronary atherosclerotic severity [6].

Taken together, these studies suggest that the association between skeletal and coronary disease is more consistently identified when coronary atherosclerosis is characterized by its extent or overall burden rather than solely by the presence of a stenosis. Differences in age, sex distribution, clinical presentation, skeletal measurement site, and angiographic definitions may further explain the variation among studies. By enrolling older adults with established chronic coronary syndrome, measuring bone mineral density at both the lumbar spine and femoral neck, and assessing coronary disease through complementary measures of vessel involvement and Gensini severity, our study adds evidence that DXA-defined osteoporosis is associated with a more advanced angiographic phenotype of coronary artery disease. In the exploratory three-category analysis, the greatest angiographic CAD burden was consistently observed among participants with osteoporosis, whereas osteopenia did not differ significantly from normal BMD across the evaluated CAD-burden measures. These findings support retaining DXA-defined osteoporosis, rather than a broader low-BMD category, as the primary exposure in the present study.

Several biological mechanisms may explain the observed association between osteoporosis and more extensive coronary artery disease. Rather than representing two independent age-related conditions, osteoporosis and atherosclerosis appear to share multiple pathophysiological pathways, including chronic low-grade inflammation, oxidative stress, endothelial dysfunction, cellular senescence, and dysregulated calcium–phosphate metabolism [20]. Aging is accompanied by increased production of pro-inflammatory cytokines, such as interleukin-6 and tumor necrosis factor-α, which stimulate osteoclast activation while simultaneously promoting endothelial injury and vascular inflammation [20]. Oxidative stress further contributes to both bone resorption and atherogenesis by impairing osteoblast differentiation, reducing nitric oxide bioavailability, and accelerating vascular aging [20]. These shared mechanisms may partly explain why osteoporosis frequently coexists with diffuse coronary atherosclerosis in older adults.

In addition, increasing evidence supports the concept of a bone–vascular axis, in which common molecular pathways regulate both bone remodeling and vascular calcification [21]. The receptor activator of nuclear factor-κB (RANK), RANK ligand (RANKL), and osteoprotegerin (OPG) signaling pathway plays a central role in skeletal homeostasis while also influencing vascular smooth muscle cell osteogenic differentiation and arterial calcification [22]. Likewise, dysregulation of Wnt/β-catenin and bone morphogenetic protein signaling has been implicated in both osteoporosis and vascular mineralization [23,24]. These observations suggest that osteoporosis may reflect a systemic biological phenotype characterized by accelerated musculoskeletal and vascular aging rather than an isolated skeletal disorder. Nevertheless, because of the cross-sectional design, our findings should be interpreted as demonstrating an association rather than a causal relationship.

The present findings have several potential clinical implications. First, osteoporosis identified by dual-energy X-ray absorptiometry may provide additional information regarding the overall burden of coronary atherosclerosis in older adults with chronic coronary syndrome. Although bone mineral density assessment should not be considered a diagnostic tool for coronary artery disease, recognition of osteoporosis may prompt clinicians to perform a more comprehensive cardiovascular risk assessment and optimize preventive strategies in this high-risk population [1]. Conversely, older adults with established chronic coronary syndrome may also benefit from routine evaluation of skeletal health, given the substantial coexistence of osteoporosis and cardiovascular disease [25]. An integrated approach addressing both cardiovascular and skeletal health may therefore be particularly relevant in geriatric practice. Given the cross-sectional design and single-timepoint assessment of BMD, our findings should not be interpreted as supporting the use of DXA to monitor CAD progression. However, prospective studies are required before osteoporosis can be incorporated into cardiovascular risk stratification algorithms [26].

This study has several strengths. To our knowledge, it is among the few studies specifically evaluating the association between DXA-defined osteoporosis and angiographic coronary artery disease burden in older adults with chronic coronary syndrome. The two-center design enhances the generalizability of the findings within this patient population. Bone mineral density was assessed using dual-energy X-ray absorptiometry, the current reference standard for the diagnosis of osteoporosis, while coronary artery disease was comprehensively characterized using both the anatomical extent of vessel involvement and the Gensini score, providing complementary assessments of disease burden. In addition, the multivariable analyses accounted for major cardiovascular risk factors, supporting the robustness of the observed associations.

Several limitations should also be acknowledged. First, the cross-sectional design precludes determination of temporal or causal relationships between osteoporosis and coronary artery disease. Second, participants were recruited from two tertiary hospitals and all underwent clinically indicated coronary angiography, which may introduce selection bias and limit the generalizability of the findings to the broader older population with chronic coronary syndrome. Third, we did not evaluate biomarkers related to bone metabolism, such as serum vitamin D, parathyroid hormone, fibroblast growth factor-23, or bone turnover markers, which may have provided additional mechanistic insights. In addition, lumbar spine bone mineral density measurements in older adults may be influenced by degenerative spinal changes or vascular calcification, potentially leading to overestimation of bone mineral density. Previous coronary revascularization may also have influenced the angiographic assessment of CAD burden. Although available angiographic and revascularization records were considered when characterizing established coronary involvement, the vessel-based measures may not fully represent untreated stenosis burden at a single timepoint, and some degree of misclassification cannot be excluded. Finally, despite adjustment for major confounders, residual confounding from unmeasured factors cannot be completely excluded.

## 5. Conclusions

In conclusion, DXA-defined osteoporosis was independently associated with a greater angiographic burden of coronary artery disease in older adults with chronic coronary syndrome. Patients with osteoporosis were more likely to have extensive coronary involvement and severe coronary atherosclerosis, independent of conventional cardiovascular risk factors. These findings demonstrate an association between osteoporosis and greater coronary artery disease burden in this population but do not establish a temporal or causal relationship. Prospective studies are warranted to clarify the temporal relationship between osteoporosis and coronary artery disease and to determine whether incorporating bone health assessment into cardiovascular risk stratification improves risk prediction and clinical outcomes.

## Figures and Tables

**Table 1:** Baseline Characteristics of the Study Population According to Osteoporosis Status.

Characteristic	Overall (*n* = 451)	No Osteoporosis (*n* = 300)	Osteoporosis (*n* = 151)	*p*-Value
Demographic and lifestyle characteristics				
Age, years	70.4 ± 7.1	69.6 ± 6.8	72.0 ± 7.4	<0.001
Male sex	297 (65.9)	240 (80.0)	57 (37.7)	<0.001
Body mass index, kg/m^2^	23.6 ± 2.9	24.0 ± 2.8	22.6 ± 2.9	<0.001
Smoking history	75 (16.6)	62 (20.7)	13 (8.6)	0.001
Alcohol consumption	21 (4.7)	18 (6.0)	3 (2.0)	0.061
Regular physical activity	333 (73.8)	236 (78.7)	97 (64.2)	0.001
Medical history and comorbidities				
Hypertension	387 (85.8)	262 (87.3)	125 (82.8)	0.191
Diabetes mellitus	155 (34.4)	106 (35.3)	49 (32.5)	0.543
Dyslipidemia	349 (77.4)	230 (76.7)	119 (78.8)	0.608
Heart failure	56 (12.4)	31 (10.3)	25 (16.6)	0.059
Chronic kidney disease	153 (33.9)	97 (32.3)	56 (37.1)	0.314
Chronic obstructive pulmonary disease	11 (2.4)	8 (2.7)	3 (2.0)	0.758
Previous fracture	50 (11.1)	26 (8.7)	24 (15.9)	0.021
Geriatric assessment				
Frailty	157 (34.8)	77 (25.7)	80 (53.0)	<0.001
Malnutrition or nutritional risk	47 (10.4)	23 (7.7)	24 (15.9)	0.007
ADL impairment	52 (11.5)	22 (7.3)	30 (19.9)	<0.001
IADL impairment	152 (33.7)	75 (25.0)	77 (51.0)	<0.001
Cognitive impairment	64 (14.2)	35 (11.7)	29 (19.2)	0.030

Data are presented as mean ± standard deviation (SD) or *n* (%), unless otherwise indicated. Continuous variables were compared using Welch’s independent-samples *t*-test. Categorical variables were compared using Pearson’s χ^2^ test or Fisher’s exact test, as appropriate. ADL, activities of daily living; IADL, instrumental activities of daily living.

**Table 2:** Coronary Angiographic Characteristics According to Osteoporosis Status.

Characteristic	Overall (n = 451)	No Osteoporosis (n = 300)	Osteoporosis (n = 151)	*p*-Value
Extent of coronary artery disease				
One-vessel disease	171 (37.9)	139 (46.3)	32 (21.2)	
Two-vessel disease	97 (21.5)	74 (24.7)	23 (15.2)	
Three-vessel disease	140 (31.0)	70 (23.3)	70 (46.4)	
Left main disease †	104 (23.1)	55 (18.3)	49 (32.5)	0.001
Overall comparison of one-, two-, and three-vessel disease				<0.001
≥2-vessel or left main disease	287 (63.6)	167 (55.7)	120 (79.5)	<0.001
≥3-vessel or left main disease	201 (44.6)	100 (33.3)	101 (66.9)	<0.001
Angiographic severity ‡				
Gensini score, median (IQR)	25 (10–49)	24 (10–44)	28 (9–59)	0.130
Mild (0–23)	124 (46.6)	84 (48.6)	40 (43.0)	
Moderate (24–54)	89 (33.5)	63 (36.4)	26 (28.0)	
Severe (>54)	53 (19.9)	26 (15.0)	27 (29.0)	
Overall comparison of Gensini severity categories				0.022

Data are presented as *n* (%) or median (interquartile range [IQR]), as appropriate. Comparisons between groups were performed using Pearson’s χ^2^ test or Fisher’s exact test for categorical variables and the Mann–Whitney *U* test for continuous variables, as appropriate. † Left main disease is presented separately because it may coexist with one-, two-, or three-vessel coronary artery disease. ‡ Gensini scores were available for 266 participants (173 without osteoporosis and 93 with osteoporosis). Percentages for the Gensini severity categories were calculated using these available-case denominators.

**Table 3:** Logistic Regression Analyses of the Association Between Osteoporosis and Coronary Artery Disease Burden.

Outcome	No Osteoporosis n/N (%)	Osteoporosis n/N (%)	Crude OR (95% CI)	*p*-Value	Adjusted OR * (95% CI)	*p*-Value
≥2-vessel or left main disease	167/300 (55.7)	120/151 (79.5)	3.08 (1.95–4.86)	<0.001	3.97 (2.31–6.81)	<0.001
≥3-vessel or left main disease	100/300 (33.3)	101/151 (66.9)	4.04 (2.67–6.12)	<0.001	5.07 (3.06–8.41)	<0.001
Severe coronary artery disease (Gensini score > 54) †	26/173 (15.0)	27/93 (29.0)	2.31 (1.25–4.26)	0.007	3.08 (1.50–6.36)	0.002

Abbreviations: OR, odds ratio; CI, confidence interval; Participants without osteoporosis served as the reference group. * Adjusted odds ratios were estimated using multivariable logistic regression models adjusted a priori for age, sex, body mass index, hypertension, diabetes mellitus, chronic kidney disease, and smoking history. † The analysis of severe coronary artery disease was restricted to the 266 participants with available Gensini scores (173 without osteoporosis and 93 with osteoporosis).

## Data Availability

The data presented in this study are available from the corresponding author upon reasonable request. The data are not publicly available because they contain information that could compromise the privacy of the study participants.
